# Ovarian epithelial cancer: a role for PGE_2_-synthesis and signalling in malignant transformation and progression

**DOI:** 10.1186/1476-4598-5-62

**Published:** 2006-11-16

**Authors:** Katarina Rask, Yihong Zhu, Wanzhong Wang, Lars Hedin, Karin Sundfeldt

**Affiliations:** 1 Department of Physiology, Sahlgrenska Academy at Göteborg University, Göteborg, Sweden; 2 Department of Clinical Sciences, Section for Obstetrics and Gynecology, Sahlgrenska Academy at Göteborg University, Göteborg, Sweden; 3 Department of Clinical Sciences, Section for Urology, Sahlgrenska Academy at Göteborg University, Göteborg, Sweden; 4 Department of Education, Weill Cornell Medical College in Qatar, Doha, Qatar

## Abstract

**Background:**

The involvement of the cyclooxygenases (COX), in particular COX-2, is well documented for many tumours, e.g. colon, breast and prostate cancer, by both experimental and clinical studies. There are epidemiological data from subjects using NSAIDs, and experimental evidence supporting the hypothesis of prostaglandins (PGs) as regulators of tumourigenesis in the ovary. One of the end products of PG-synthesis, PGE_2_, regulates several key-processes, which are characteristic for tumour growth, e.g. angiogenesis, proliferation and apoptosisis. The present study investigated the pathway for PGE_2 _– synthesis and signalling in ovarian tumourigenesis by analysing specimen from normal ovaries (n = 18), benign (B) (n = 8), borderline type (BL) (n = 6) and malignant tumours (AC) (n = 22). The expression and cell-specific localization of COX-1, COX-2, microsomal prostaglandin E synthase-1 (mPGES-1) and two of the receptors for PGE_2_, EP_1 _and EP_2_, were examined by immunoblotting (IB) and immunohistochemistry (IHC).

**Results:**

The results are in line with earlier studies demonstrating an increase of COX-2 in AC compared to the normal ovary, B and BL tumours. Increased expressions were also observed for COX-1, mPGES-1 and EP-1 which all were significantly (p < 0.05) augmented in less differentiated AC (grades: moderately-, poorly- and undifferentiated). The increase of COX-2 was also correlated to stage (FIGO classification) with significant elevations in stages II and III. EP_1 _was increased in stage III while no significant alterations were demonstrated for COX-1, mPGES-1 or EP_2 _for stage. IHC revealed staining of the tumour cells, but also increase of COX-1, COX-2, mPGES-1 and EP_1–2 _in the stromal compartment of AC (grades: moderately-, poorly- and undifferentiated). This observation suggests interactions between tumour cells and stromal cells (fibroblasts, immune cells), e.g. paracrine signalling mediated by growth factors, cytokines and possibly PGs.

**Conclusion:**

The increases of COX-1, COX-2, mPGES-1 and EP_1–2 _in epithelial ovarian cancer, supports the hypothesis that PGE_2_-synthesis and signalling are of importance for malignant transformation and progression. The observed augmentations of COX-1, COX-2 and mPGES-1 have implications for future therapeutic strategies.

## Background

Epithelial ovarian cancer (EOC), which compromises 90% of all ovarian malignancies, is the leading cause of death from gynaecological cancer in the western world [1]. The pathology underlying this disease is not fully understood, but an inflammatory process is one factor suggested to participate in tumourigenesis [2]. Chronic inflammatory conditions caused by talc or asbestos exposure, endometriosis or other pelvic inflammatory diseases are related to an increased incidence of EOC [3]. Several classical mediators of inflammation participate in the ovulatory process, proposing each ovulation to have resemblance with a localized inflammatory reaction [4]. The importance of inflammation/ovulation in ovarian tumourigenesis is further supported by the observed reduction in the relative risk to develop EOC in women with a decrease in the total number of ovulations during a lifetime, i.e. due to childbearing or the use of contraceptive pills [5]. This was already suggested three decades ago by Fathalla [6] as the "incessant ovulation hypothesis". Tubal ligation and hysterectomy without oophorectomy, which reduces local inflammation, also decreased the risk to develop EOC, while inflammatory conditions in the pelvis, e.g. endometriosis, were associated with increased risk of EOC [5].

A measure to evaluate the role of inflammation in tumourigenesis is to examine the correlation between the use of non-steroidal anti-inflammatory drugs (NSAIDs) and the risk to develop cancer. The use of NSAIDs or aspirin causes a reduction in the risk to develop colorectal- (CRC) and to a less extent, prostate- and breast cancer [7]. Inconsistent results have been published for EOC, but several studies suggest an inverse relationship between the use of NSAIDs and the incidence of this malignancy [8]. Several *in vivo *[9] and *in vitro *[10-13] experiments have demonstrated reduced tumour growth [9], decreased proliferation [11], cell cycle arrest [10], attenuated metalloprotease-dependent motility and invasive activity [13], and reduced expression of vascular endothelial growth factor expression [14] by NSAIDs in models for EOC.

COX-1 and COX-2 are key enzymes in the synthesis of prostanoids by converting arachidonic acid to PGG_2 _and subsequently to PGH_2 _[15]. PGE_2 _is derived from PGH_2 _by a synthase, prostaglandin E synthase (PGES). Three isoforms of PGES have been identified, microsomal prostaglandin E synthase (mPGES-1 and mPGES-2) and cytosolic PGES (p23) [15]. Prostaglandin E_2 _(PGE_2_) was implicated in tumour progression [15] and increased contents of prostanoids (PGE_2 _and TxB_2_) in ovarian tumours have been described [16,17]. Later studies have reported PGE_2 _as a regulator of proliferation and apoptosis in ovarian cancer cell lines [18]. Several reports have shown that either COX-1 or COX-2 is up-regulated in EOC [14,19-21]. Ovarian tumours with increased content of COX-2 were associated with chemotherapy resistance and poor prognosis [22] while COX-1 was suggested to participate in neo-vascularization [14].

PGE_2 _is ligand for at least four membrane-bound receptors, EP_1–4_. EP_2 _was induced in colon polyps in mice with a targeted deletion of the adenomatosis polyposis coli (APC) gene [23] and this receptor has also a key role in the ovulatory process [24]. Studies in EP_1 _receptor knockout mice and experiments with selective receptor antagonists, have demonstrated a role for this receptor in colon and breast carcinogenesis [25]. PGE_2 _is produced by a variety of cells and has a broad range of effects in individual cells and organs.

We choosed to analyse the rate-limiting enzymes (COX-1, COX-2 and mPGES-1) for PGE_2_-synthesis and two of its receptors (EP_1–2_) in specimen from normal ovaries and from epithelial ovarian tumours of different grades and stages, as an approach to delineate the role for PGE_2 _in ovarian tumorigenesis, since the prostanoid itself is quickly redistributed and degraded in whole tissue biopsies. Furthermore, the combined techniques of immunoblotting and immunohistochemistry allowed us to determine the contents as well as the cell-specific localizations of the individual proteins. The cell- and stage specific increases of COX-1, COX-2, mPGES-1 and EP_1–2_, support the hypothesis that PGE_2_-synthesis and signalling are of importance for malignant transformation and progression in EOC. The observed augmentations of PGE_2_-signalling capacity have implications for future therapies.

## Materials and methods

### Human tissues

Biopsies from normal human ovaries were obtained from 18 women undergoing laparotomy or laparoscopy for non- ovarian diseases/benign pelvic conditions. Ovarian epithelial tumours tissues were obtained from 36 women (approved by the Ethics Committee of the Medical Faculty, Göteborg University) (Table 1). Tissues were immediately washed in ice-cold saline, snap-frozen in liquid nitrogen and stored at -70°C until analysis [26,27]. All samples were examined by an experienced pathologist and classified according to FIGO for diagnosis.

**Table 1:** Histological description

*Sample no*.	*P-H*	*Sample no*.	*P-H*	*Grade*	*Stage*
1	NF	27	BLS		I
2	NF	28	BLS		IB
3	NF	29	BLS		IA
4	NF	30	BLM		IA
5	NF	31	BLM		IIB
6	NF	32	BLM		II
		
7	NF	33	AS	High	II
8	NF	34	AM	High	IB
				
9	NP	35	AS	High/Moderate	III
10	NP	36	AS	High/Moderate	I
11	NP	37	AS	Moderate	III
12	NP	38	AS	Moderate	III
13	NP	39	AS	Moderate	III
14	NP	40	AS	Moderate	II
15	NP	41	AS	Moderate	II
16	NP	42	AS	Poor	III
17	NP	43	AS	Poor	III
18	NP	44	AS	Poor	III
				
19	BS	45	AS	Poor	III
20	BS	46	AS	Poor	III
21	BS	47	AS	Poor	III
22	BS	48	AS	Poor	III
23	BM	49	AS	Poor	II
24	BM	50	AS	Poor	II
25	BM	51	AM	Poor	III
26	BM	52	AM	Poor	III
				
		53	AU	Undiff	III
		54	AU	Undiff	I

Histological description of ovarian tissue including sample numbers which can be found in representative immunoblots and immunohistochemistry figures. Abbreviations: P-H; patho-histologic description, NF; normal fertile, NP; normal postmenopausal, BS; benign serous, BM; benign mucinous, BLS; borderline type serous, BLM; borderline type mucinous, AS; adenocarcinoma serous, AM; adenocarcinoma mucinous, AU; adenocarcinoma undifferentiated type.

### Immunoblotting

Soluble tissue extracts were prepared from 45 tissue biopsies (Table 1), and immunoblotting was performed as previously described [26,27]. Thirty-five μg of total protein was loaded into each well for 1D SDS PAGE (4–12% Bis-Tris NuPage gels, Invitrogen, Paisley, UK). The blotting-membranes were incubated with blocking buffer containing the following primary antibodies from Cayman Chemical Co. (Ann Arbor, MI, USA): COX-1 (cat. no. 160110, monoclonal, dilution 1:1,000), COX-2 (cat. no. 160126, rabbit polyclonal, dilution 1:1,000), mPGES-1 (cat. no. 160140, rabbit polyclonal, dilution 1:500), EP_1 _receptor (cat. no. 101740, rabbit polyclonal, dilution 1:500) and EP_2 _receptor (cat. no. 101750, rabbit polyclonal, dilution 1:500). COX-1 and COX-2 electrophoresis standards from Cayman Chemical Co. (cat. no. 360100, 360120) were used as positive controls. The specificity of the COX-2 antibody was verified by the addition of a blocking peptide to the antibody solution prior to immunodetection (Cayman Chemical Co,. cat. no. 360106). Ovaries obtained from immature rats stimulated with pregnant mare serum gonadotrophin (PMSG) and human chorionic gonadotropin (hCG), (preovulatory ovaries) were used as positive control for mPGES-1 and EP_1–2_. Immunoreactive proteins were visualized by chemiluminescence using alkaline-phosphatase-conjugated secondary goat anti-mouse or goat anti-rabbit antibodies (dilution 1:40 000), and the CDP-Star^® ^substrate (Tropix, Bedford, UK). The conditions were kept constant during the analysis of the immunoblots and each blot contained an internal control sample (see below and Figure legend 1 for details) in order to compare the levels of expression between blots [26,27].

### Immunohistochemistry

Frozen biopsies from 18 normal ovaries and ovarian tumours (Table 1) were cryosectioned (5–7 μm) and mounted on electrically charged glass slides, fixed in cold acetone at -20°C for 10 min and air dried in room temperature. The endogenous peroxidase activity was blocked by treatment for 4 min with peroxidase block from DAKO EnVision™+System, Peroxidase (AEC) kit (DAKO Corporation, USA). Primary antibodies against COX-1 (1:100), COX-2 (Cayman Chemical Co., cat. no. 160112, monoclonal, 1:100), mPGES-1 (1:50), EP_1 _(1:50) EP_2 _(1:50) and CK8 (1:400) diluted in TBS were applied overnight at 4°C, followed by biotinylated goat anti-mouse immunoglobulin (1:60) (VECTASTAIN^® ^ABC kit, Vector Laboratories, Inc., Burlingame, CA) for 45 min at 37°C and finally avidin-biotin-peroxidase complex (VECTASTAIN^® ^ABC kit, Vector Laboratories) at 37°C for 30 min. The enzymatic reaction was developed using AEC Chromogen Substrate Buffer (DAKO EnVision™+System, Peroxidase (AEC) kit, DAKO). Slides were counterstained with haematoxylin and mounted in 10% glycerol. The monoclonal COX-2 antibody used here was evaluated previously for immunohistochemistry by blocking experiments using a specific peptide [28]. Antibodies against cytokeratin 8 (CK8) (monoclonal, Low Molecular Weight, DAKO, Copenhagen, Denmark), was used as a marker for epithelial cells. Primary antibodies were replaced by equal amounts of TBS for negative controls. The sections were viewed in a Nikon microphot FX microscope and photographed with a Nikon Coolpix 990 digital camera.

### Densitometric scanning

Semi-quantitative measurements of proteins from the immunoblots were made by densitometry (Fluor-S™ Multimager, Quantity One ver. 4.1.0., BioRad, Hercules, CA, USA). The optical density (OD) of each band was measured. An ovarian sample was used as an internal standard on each blot, and the measured value of this sample was set to 100%. The signal from each band was then compared to the standard and the obtained relative value was used for statistical analysis [26,27].

### Statistics

Values are given as mean ± SEM. ANOVA followed by Fischer's LSD post-hoc test was used for statistical analysis of the data obtained by densitometric scanning. A p-value less than 0.05 was considered to be significant. Data is presented in a histogram and with a typical immunoblot of selected samples for each analysed protein.

## Results

### Increases of COX-1, COX-2, mPGES-1 and EP_1 _in adenocarcinomas

**Figure 1:**
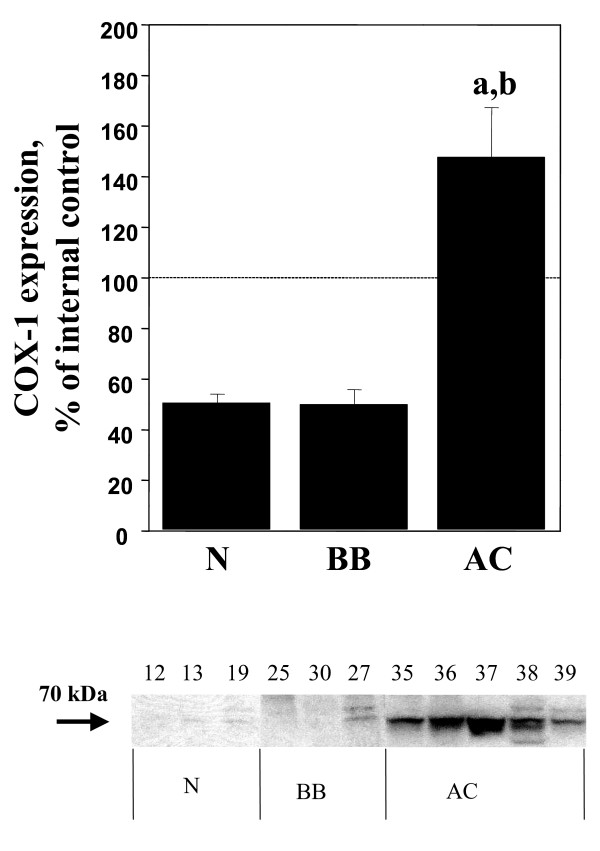
Relative protein expressions (mean ± SEM) of COX-1 in normal ovaries (N = pre- and postmenopausal ovaries (n = 10), BB = benign- and borderline type tumours, (n = 13), and AC = adenocarcinomas (n = 22).**(a) **P < 0.05 vs normal samples (N), **(b) **P < 0.05 vs benign/borderline (BB). The measurements are presented as percentage changes compared to a reference sample (tumour tissue). Below each histogram a representative immunoblott is presented. Each number correlates to the patho-histological description of the sample (see Table 1).

**Figure 2:**
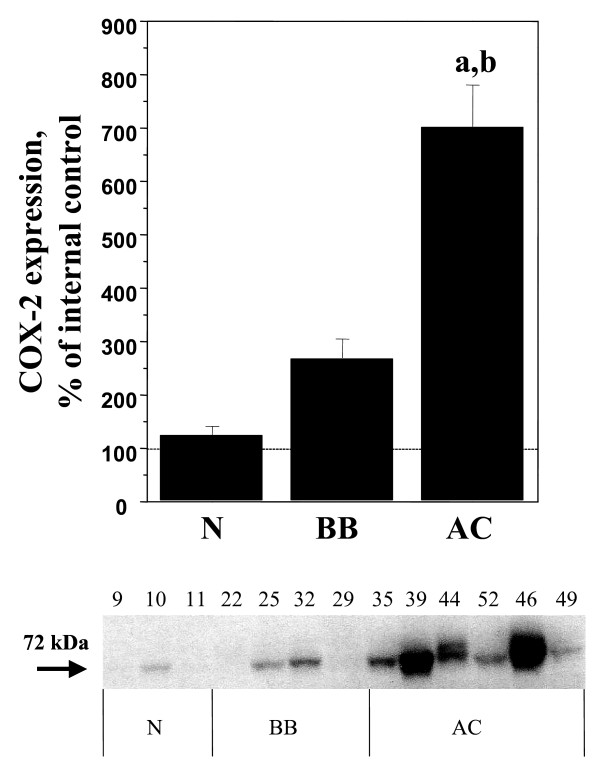
Relative protein expressions (mean ± SEM) of COX-2 in normal ovaries (N = pre- and postmenopausal ovaries (n = 10), BB = benign- and borderline type tumours, (n = 13), and AC = adenocarcinomas (n = 22).**(a) **P < 0.05 vs normal samples (N), **(b) **P < 0.05 vs benign/borderline (BB). The measurements are presented as percentage changes compared to a reference sample (normal ovarian tissue). Below each histogram a representative immunoblott is presented. Each number correlates to the patho-histological description of the sample (see Table 1).

**Figure 3:**
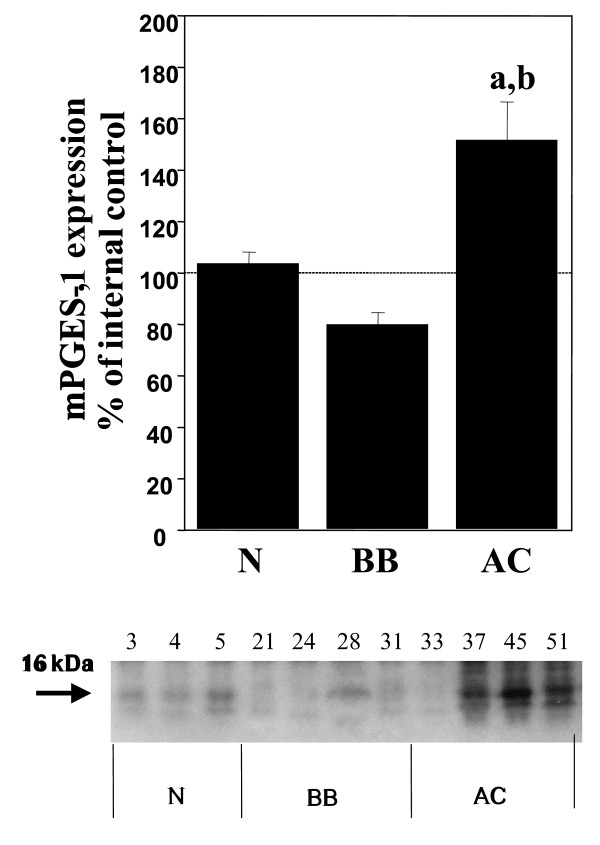
Relative protein expressions (mean ± SEM) of mPGES-1in normal ovaries (N = pre- and postmenopausal ovaries (n = 10), BB = benign- and borderline type tumours, (n = 13), and AC = adenocarcinomas (n = 22).**(a) **P < 0.05 vs normal samples (N), **(b) **P < 0.05 vs benign/borderline (BB). The measurements are presented as percentage changes compared to a reference sample (normal ovarian tissue). Below each histogram a representative immunoblott is presented. Each number correlates to the patho-histological description of the sample (see Table 1).

**Figure 4:**
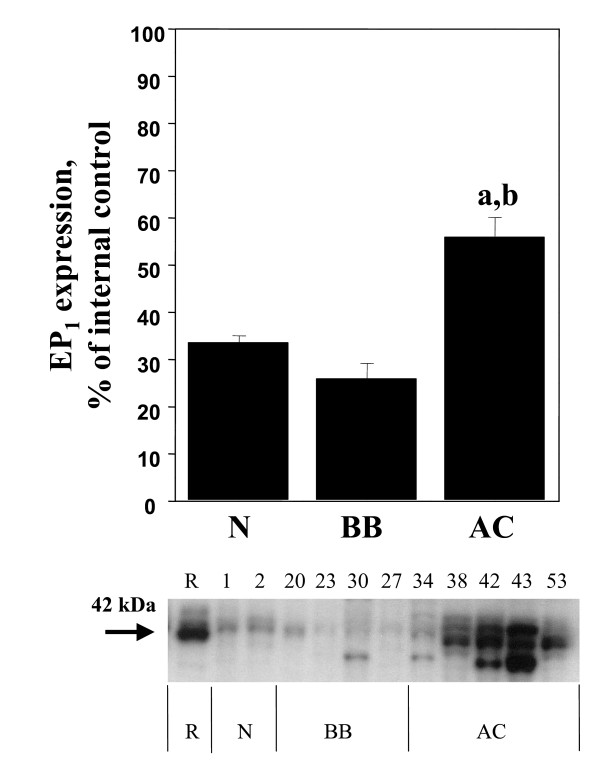
Relative protein expressions (mean ± SEM) of EP_1 _in normal ovaries (N = pre- and postmenopausal ovaries (n = 10), BB = benign- and borderline type tumours, (n = 13), and AC = adenocarcinomas (n = 22).**(a) **P < 0.05 vs normal samples (N), **(b) **P < 0.05 vs benign/borderline (BB). The measurements are presented as percentage changes compared to a reference sample (rat preovulatory ovary). Below each histogram a representative immunoblott is presented. Each number correlates to the patho-histological description of the sample (see Table 1).

**Figure 5:**
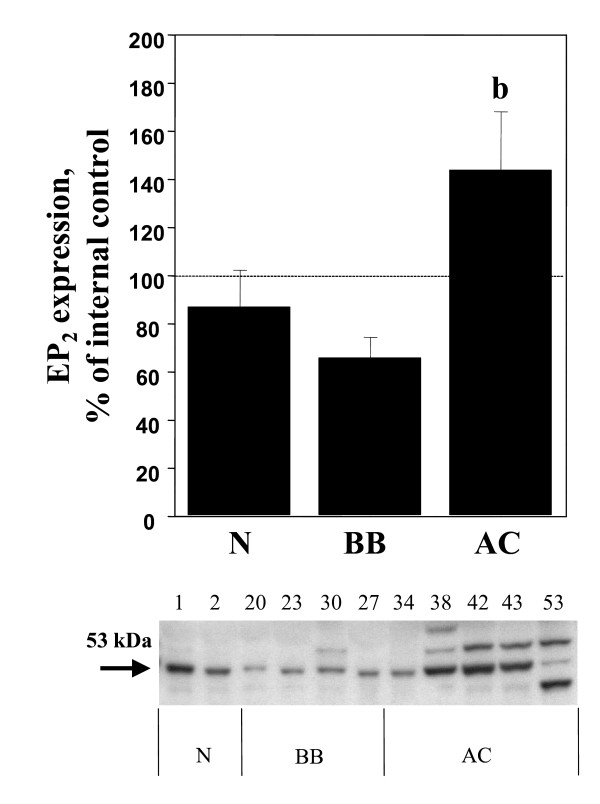
Relative protein expressions (mean ± SEM) of EP_2 _in normal ovaries (N = pre- and postmenopausal ovaries (n = 10), BB = benign- and borderline type tumours, (n = 13), and AC = adenocarcinomas (n = 22).**(b) **P < 0.05 vs benign/borderline (BB). The measurements are presented as percentage changes compared to a reference sample (normal ovarian tissue). Below each histogram a representative immunoblott is presented. Each number correlates to the patho-histological description of the sample (see Table 1).

Significant (p < 0.05) increases of COX-1, COX-2, mPGES-1 and EP_1 _were demonstrated in the adenocarcinomas (AC) in comparison with normal ovarian tissue, benign adenomas and borderline tumours (B/BL) (Figure 1, 2, 3, 4, 5). EP_2 _was also increased in comparison to the B/BL tumours. An increase, although not significant, of COX-2, was noticed in the B/BL group, compared to specimens from normal ovaries.

**Figure 6:**
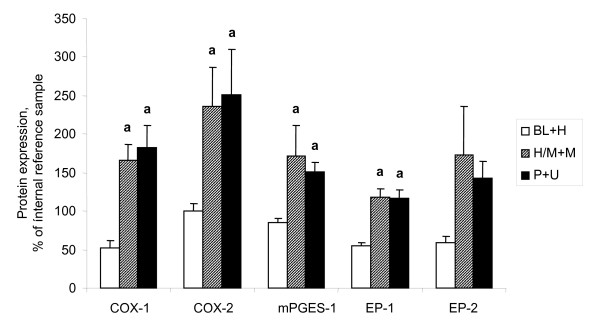
Relative expressions (mean ± SEM) of COX-1, COX-2, mPGES-1, EP_1 _and EP_2_. Tissue contents in tumours related to grade of differentiation. (BL+H = borderline type tumours and highly differentiated tumours (n = 8), H/M+M = highly to moderately, and moderately differentiated tumours (n = 7), P+U = poorly and un-differentiated tumours (n = 13).**(a) **P < 0.05 vs borderline type tumours and highly differentiated tumours (B/BL), **(b) **P < 0.05 vs highly to moderately and moderately differentiated tumours (H/M+M).

**Figure 7:**
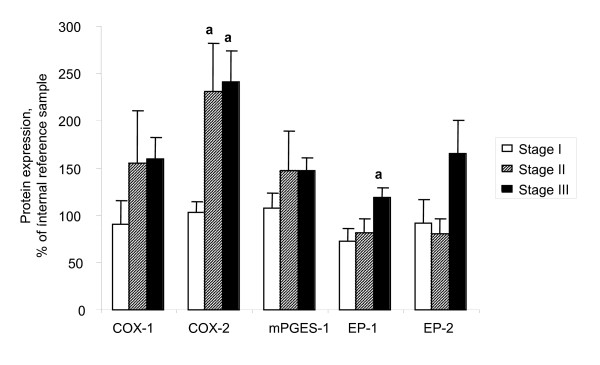
Relative expressions (mean ± SEM) of COX-1, COX-2, mPGES-1, EP_1 _and EP_2_. Tissue contents in malignant tumours (adenocarcinomas) related to stage. Stage I (n = 7), stage II (n = 6) and stage III (n = 14).**(a) **P < 0.05 vs stage I, **(b) **P < 0.05 vs stage II.

To examine if the pattern of expression was influenced by the degree of differentiation (grade) in the malignant group of tumours (AC), these tumours were dived into three groups; i) borderline tumours and highly differentiated AC (BL+H); ii) high/moderately and moderately differentiated AC (H/M+M); and iii) poorly differentiated and undifferentiated AC (P+U). The rationale to include highly differentiated AC (H) with the borderline (BL) type tumours was that these tumours are clinically considered to have more benign prognosis. The expressions of COX-1, mPGES-1 and EP_1 _were all significantly elevated in both the H/M+M and P+U group compared to the more differentiated tumours in the BL+M group. (Figure 6). There was also an increase of EP_2 _in the same groups, although not significant. COX-2 expression differed between tumours in the same histological group, but the majority of the tumours exhibited a strong signal for COX-2 (18/22 of the AC, 21/36 of all tumours), while a few tumours demonstrated a weak band (4/22 of the AC, 15/36 of all tumours). Amongst the four AC with low levels of expression, one was highly differentiated (H), two were in the group of high/moderately differentiated (H/M) and one was moderately differentiated (M). The expression patterns were further analyzed in the AC according to FIGO-stage (Figure 7). COX-2 demonstrated significant increases in both stage II and III cancers, compared to stage I. An increase was also shown for EP_1 _in stage III tumours compared to stage I. No differences were observed for COX-1, mPGES-1 or EP_2_.

A representative blot from each protein analyzed is presented in Figure 1, 2, 3, 4, 5. The major band for all the examined proteins migrated at their expected molecular weights (M_r_). For mPGES-1, EP_1_, and EP_2_, additional bands with higher and lower M_r _were observed in the group of malignant tumours (AC) (Figure 3, 4, 5). The additional M_r _species most likely represents phosphorylated forms of mPGES-1 and EP_1–2_. However, these bands for EP_1–2 _might also represent splice variants of the receptors [29].

### Cell-specific localisation of COX-1, COX-2, mPGES-1, EP_1 _and EP_2 _in the normal ovary

**Figure 8:**
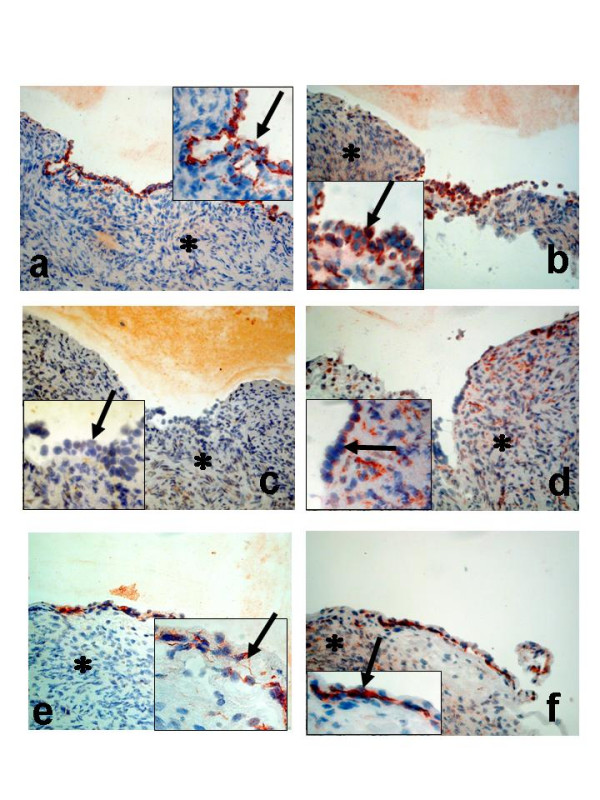
Immunohistochemical analysis of normal ovaries; (**a-f**) Postmenopausal ovary with surface epithelium; stained with antibodies against CK8 (**a**); COX-1 (**b**); COX-2 (**c**); mPGES-1 (**d**);EP_1 _(**e**); EP_2 _(**f**). *Arrow *= epithelial cells, *Star *= stroma cells. (Original magnification ×200, inserted pictures X400).

**Figure 9:**
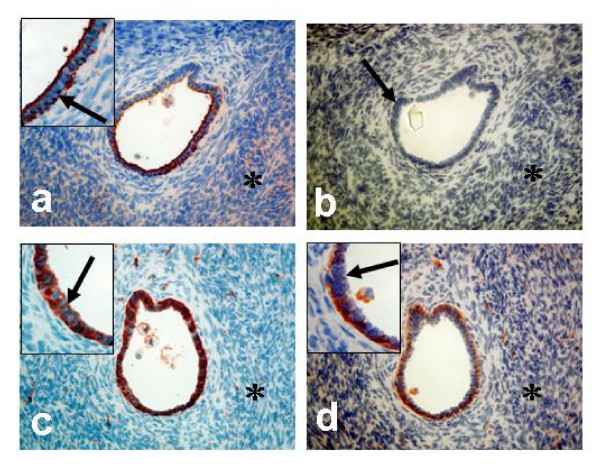
Immunohistochemical analysis of normal ovaries; (**a-d**)Premenopausal ovary with epithelial cells lining an inclusion cyst; stained with antibodies against CK8 (**a**); COX-1 (**c**); COX-2 (**d**); negative control (**b**). *Arrow *= epithelial cells, *Star *= stroma cells. (Original magnification X200, inserted pictures ×400).

Tissue biopsies from nine normal ovaries were used for immunohistochemistry. Four of the ovaries were from fertile women and five from post-menopausal women undergoing surgery for benign non-ovarian diseases. Each ovary was consecutively sectioned and stained for cytokeratin 8 (CK8), a marker for epithelial cells (Figure 8A, 9A) and the individual proteins in the PGE_2 _synthesis- and signalling pathway. DAPI was used for nuclear staining.

COX-1 was present in both surface epithelial cells (OSE) and epithelial cells lining the inclusion cysts (Figure 8B, 9C). Some staining was also observed in the stroma (Figure 8B). The signal for COX-2 in the OSE was negligible. However, weak staining was noticed in some areas of the underlying stroma (Figure 8C). An interesting observation was the clear staining for COX-2 in the epithelial cells of the inclusion cysts (Figure 9D). Staining for mPGES-1 was predominantly localized to cells in the stroma. Some of the epithelial cells on the ovarian surface demonstrated staining for mPGES-1 (Figure 8D). Both EP_1 _and EP_2 _were expressed in OSE, while only EP_2 _was present in stroma cells, an expression pattern similar to that observed for mPGES-1 (Figure 8E,F).

### Cell-specific localisation of COX-1, COX-2, mPGES-1, EP_1 _and EP_2 _in benign, borderline and malignant ovarian tumours

**Figure 10:**
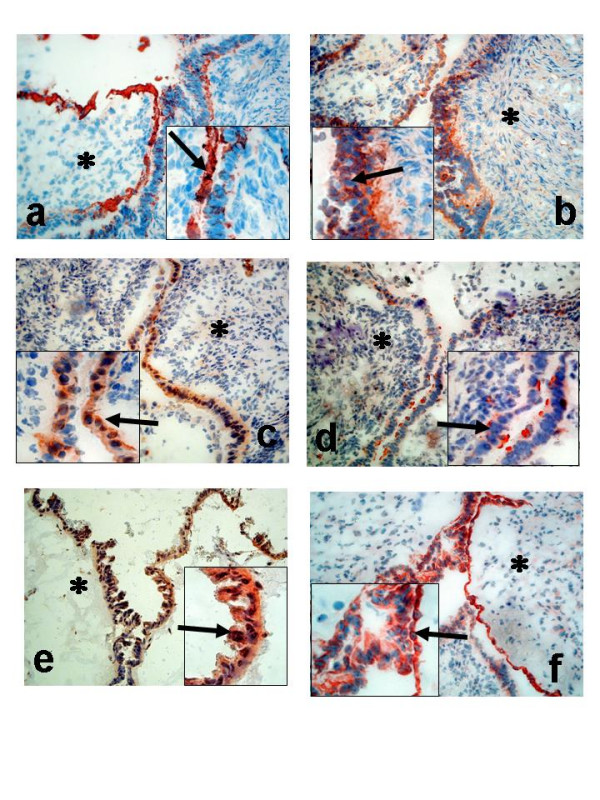
Immunohistochemical analysis of ovarian tumours; Serous borderline type tumour, stage I (**a-f**); Staining with antibodies against CK8 (**a**); COX-1 (**b**); COX-2 (**c**); mPGES-1 (**d**);EP_1 _(**e**); EP_2 _(**f**). *Arrow *= epithelial cells, *Star *= stroma cells. (Original magnification ×200, inserted pictures ×400).

**Figure 11:**
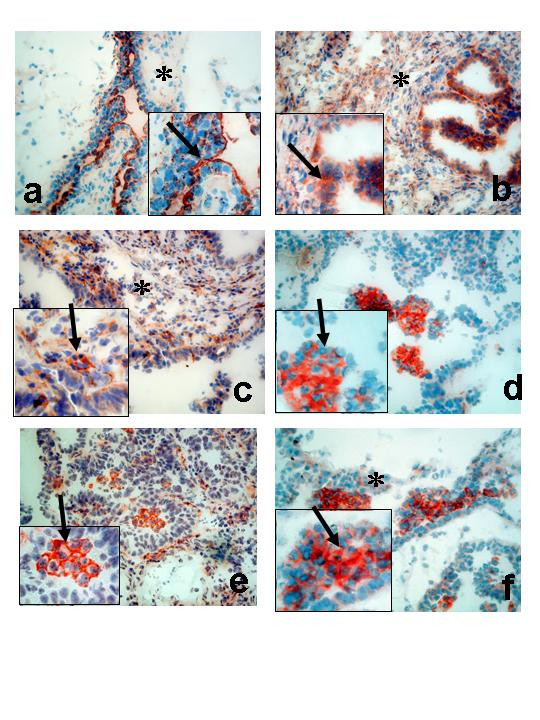
Immunohistochemical analysis of ovarian tumours; Serous adenocarcinoma, poorly differentiated, stage II (**a-f**). Staining with antibodies against CK8 (**a**); COX-1 (**b**); COX-2 (**c**); mPGES-1 (**d**);EP_1 _(**e**); EP_2 _(**f**). *Arrow *= epithelial cells, *Star *= stroma cells. (Original magnification ×200, inserted pictures ×400).

Five benign cystadenomas (three serous and two mucinous), three BL (two serous and one mucinous), and three serous ovarian AC (one highly, one moderately and one poorly differentiated AC) were used for immunohistochemistry (IHC). Serial sections from each tumour were stained for the expression of COX-1, COX-2, mPGES-1, EP_1 _and EP_2_. CK8 was used as a marker for epithelial derived cells (Figure 10A, 11A). The results are presented as representative pictures from a serous borderline type tumour (stage I, Figure 10A–F) and a poorly differentiated serous AC (stage II, Figure 11A–F).

Distinct staining of COX-1 was demonstrated mainly in the epithelial cells of the BL tumour (Figure 10B). COX-2 exhibited an even more epithelial concentrated staining compared to COX-1 (Figure 10C). Both COX-1 and COX-2 was confined to the cytoplasm of the epithelial cells (inserts, Figure 10B–C). Microsomal PGES-1 was expressed predominantly in epithelial cells, but to some degree also in the stroma, although the staining was much less homogenous compared to COX-1 and COX-2 (Figure 10D). EP_1 _and EP_2 _demonstrated almost identical staining patterns with both receptors localised to the epithelial cells of the tumour (Figure 10E,F).

The staining pattern for the AC was different compared to that of the BL tumours. One exception was COX-1, which was still predominantly localised to the epithelial cells, some expression was also present in the stroma (Figure 11B). An increased stromal content was also noticed for COX-2 (Figure 11C). Intense staining was observed for mPGES-1, EP_1 _and EP_2 _in clusters of tumour cells (Figure 1111D–F).

### Discussion

Numerous experimental models have convincingly demonstrated that the increased production of PGE_2 _in various tumours, including EOC, is involved in tumorigenesis [19]. The reduced incidence of tumours by NSAIDs, as shown in epidemiological studies, supports the concept of the PGE_2 _signalling pathway as an important factor in tumour formation and growth [7]. The present study demonstrates, for the first time, increased contents and cell-specific localisation of mPGES-1 and the PGE_2 _receptors EP_1–2 _in EOC of different grades and stages compared to normal ovaries. The study also confirms the earlier demonstrated expression patterns of COX-1 [14,21] and COX-2 in EOC [19].

The ovulatory process exhibits several signs related to an inflammatory reaction, e.g. hyperaemia, extravasation of leukocytes, proteolytic- and collagenolytic activity [4,30]. One of the key mediators in inflammation, the prostanoids, are also released during ovulation from the ovulating follicle and surrounding ovarian stroma, together with other autocrine/paracrine factors [31]. In the human ovary, COX-2 is present in the ovulating follicle during the preovulatory phase, followed by interstitial localisation of the enzyme after ovulation [32]. These prostanoids, in particular PGE_2_, are thought to contribute to proliferation and repair of OSE at the site of rupture, in addition to their crucial role(s) in the intrafollicular regulation of the ovulatory process [30]. Repetitive trauma to the epithelial cell surface, followed by exposure to mitogenic factors, is suggested to be an initiating event in ovarian tumourigenesis [6,31]. When the EOC was established, elevated levels of the prostacyclins (PGI_2_) and tromboxane (TxA_2_) in plasma, as well as increased tumour contents of PGE_2 _and TxB_2 _were reported [16,17]. Furthermore, ovarian tumours with high tissue contents of PGE_2_, PGF_2_α and 6-keto-PGF_1_α demonstrated a reduced response to chemotherapy [33].

COX-1 is constitutively expressed and plays a "housekeeping role" in many cells and has previously been demonstrated in normal OSE and in inclusion cysts [34] which are in line with the results of the present study. Furthermore, the contents of COX-1 was unaltered in the group of benign and borderline type tumours compared to normal ovaries, and the cellular localisation remained confined to the epithelial cells of these tumours. An increase of COX-1 was significant in the malignant tumours, but only in the less differentiated groups (H/M+M and P+U). COX-1 staining was no longer limited to the epithelial tumour cells since localisation to the underlying stroma also was observed. No correlation was found between FIGO stage and COX-1. Earlier studies have also reported an increase of COX-1 at both the mRNA and protein levels in EOC [14]. A stimulatory effect on neo-vascularisation was proposed since a correlation was demonstrated between COX-1 expression and the expressions of angiogenic factors (VEGF, HIF-1α, Flk1) [14]. Studies by Spinella and coworkers [12] demonstrated that endothelin (ET-1) increased COX-1 in ovarian carcinoma cells *in vitro*. Since ET-1 is known regulator of VEGF, this effect might be mediated by COX-1/PGE_2 _in these cells. Interestingly, several studies on ovarian cancer cell lines have emphasised the contribution of COX-1 to ovarian tumourigenesis. In fact, COX-1 was the predominant isoform reported to be expressed [20,34,35]. A recent study [9] used mouse EOC cells (deletion of p53, or overexpression of c-myc/K-ras or c-myc/Akt) to demonstrate increased expression of COX-1 in these genetically engineered EOC cells. The conclusion, in accordance with the study by Kino and co-workers [35], was that COX-1 is the major regulator of PGE_2 _synthesis *in vitro*.

The observation in the present study of COX-2 as more or less absent from normal OSE confirms results reported earlier [21,34,36,37]. A clear staining for COX-2 was first observed in the inclusion cysts, and some, although not significant, elevation was noticed in the B/BL group. Li et al. [34] reported a significantly higher expression level of COX-2 in BL compared to benign tumours (B). The majority of the analysed (by IHC) tumours in their material were BL (69 BL, 18 B and 27 AC), which potentially influenced their results. They found, however, that the content was lower in BL compared to AC, which is in line with the present study. The AC demonstrated a clear elevation of COX-2, in particular in tumours of lower grades (H/M+M, P+U) in the present study. In contrast to COX-1, a significant difference with elevated levels of COX-2 was also found for later stages of tumours (stages II-III). To our knowledge, this study is the first to quantify the COX-2 protein expression by immunoblotting in ovarian tumours. Several earlier studies have shown by IHC that COX-2 is up-regulated in ovarian neoplasm using different scoring systems of staining intensity [20]. Our results confirm the IHC data on COX-2 shown by others. The observed increase of COX-2 expression in EOC was correlated to poor differentiation, shorter time to tumour progression, resistance to chemotherapy, poor prognosis and significantly shorter survival time compared to patients with tumours staining negative for COX-2 [22,36,38], suggesting COX-2 to be an independent prognostic factor [36]. COX-2 expression was also significantly correlated with microvessel density and/or VEGF expression in advanced-stage ovarian serous carcinoma [37,39,40]. In our study, absence of or weak COX-2 expression was noticed in the majority of the B/BL tumours and in only four AC, all of them of high to moderately differentiated grades, and presumably better prognosis.A recent study describing positive COX-2 staining of preneoplastic OSE adjacent to COX-2 negative neoplastic cells, presumably of higher grades [41], supports our findings. A correlation to histological subtype was not found for COX-2 in the present study. The cell-specific localisation of COX-2 in the B/BL group was almost exclusive in the epithelial cell, similar to that of COX-1.

Interestingly, both COX-1 and COX-2 were expressed in OSE lining inclusion cysts in the present study. This observation might suggest that the appearance of COX-2 in these cells is an early sign of an altered phenotype (mesothelial to epithelial transition, [31]) with malignant potential. We have previously shown that the expressions of the adhesion molecules E-cadherin [42,43] and claudin-3/4 [27] and the transcription factor C/EBPβ[44] correlated with malignancy in EOC. These proteins were also expressed in epithelial cells of inclusion cysts. Notably, the expression of C/EBPβ is a putative requirement for the regulation of COX-2 expression[45]. Li and co-workers [34] reported also the expression of COX-1 in OSE and inclusion cysts of the normal ovary, but both these two locations lacked COX-2 expression. Interestingly, in two independent studies COX-2 was not expressed in any of the ovarian cancer cell lines used(nine [34] and five [41]), although Denkert and co-workers [36] showed such expression in cell lines included in their study. The difference in COX-2 expression in cell-lines and tissue/tumour biopsies is further strengthened since positive COX-2 staining was more often found at the advancing margin of tumour invasion and new metastatic lesion [34]. An explanation to the findings of ovarian cancer cells in cell culture, not expressing COX-2, can be that cell lines are devoid of their basement membranes as well as the surrounding and supporting stroma cells. Recent studies have demonstrated important roles for stromal cells in tumourigenesis [46]. An autocrine- paracrine regulatory pathway was described for breast carcinoma and surrounding stromal cells, involving growth factors and cytokines [46]. Zhou and co-workers [47] demonstrated that stromal-epithelial interactions resulted in induction of C/EBPβ in malignant breast epithelial cells. One possibility is therefore that the expression of COX-2 in early stages of cellular transformation, e.g. in inclusion cyst formation, might be initiated by paracrine factors produced by the stromal cells, resulting in increased transcription, e.g. induced by C/EBPβ, of the *cox2 *gene. The *cox2 *promotor has previously been suggested to be of potential value for gene therapy in EOC, due to its tumour-specific activation in ovarian cancer cell lines *in vitro *[48]. Furthermore, studies on ovaries from women undergoing prophylactic oophorectomy (due to mutations in BRCA1, BRCA2, MSH1 or MLH1) demonstrated that the increase of COX-2 correlated to loss of epithelial basement membrane [49]. Additional studies are needed to establish the nature of interactions between epithelial cells in inclusions cyst and the surrounding basement membrane and stromal environment. Furthermore, the co-expression of COX-1 and COX-2 in epithelial cells of inclusion cysts and B/BL tumours, opens also the possibility fort an autocrine/paracrine regulation of the two COX isoforms by PGE_2_.

The expression and regulation of mPGES-1 share many similarities with that of COX-2, e.g. induced by pro-inflammatory stimuli and hormones [50]. This is in contrast to the two other known members of this family, mPGES-2 and cPGES, which are constitutively expressed in various cells and tissues [50]. Both these isoforms are functionally closer linked to COX-1, while mPGES-1 has a marked preference to COX-2 [50]. Staining of the normal ovary for mPGES-1 revealed expression in the stroma, while the OSE cells were mostly negative. This pattern was also observed in the BL with some staining of epithelial cells or cells associated to the epithelium. A significant increase was observed in the AC, but not in B/BL. This increase was correlated to grade, but not to stage. Intense staining was demonstrated in a cluster-like pattern, predominantly in the tumour stroma. This might to some extent reflect the presence of immune cells recruited to the tumour site as a part of an inflammatory reaction. A local, anti-inflammatory response to IL-1α was suggested [51] to involve production of cortisol in the normal OSE by an increase of 11β-HSD, while this enzyme was not expressed in cell lines derived from EOC [52]. Infiltration of macrophages into EOC was described by Klimp and co-workers [53] and their study demonstrated increased levels of COX-2 in tumour-associated macrophages. The role of mPGES-1 for PGE_2 _production in these cells are essential since isolated peritoneal macrophages from mPGES-1 null mice produced minimal amounts of this prostanoids in response to pro-inflammatory stimuli (LPS) [50]. Similar results were obtained with macrophages from C/EBPβ-deficient mice as a result of ablated mPGES-1 expression. This emphasise the role of inflammation in EOC since C/EBPβ has been demonstrated to be major regulator of pro-inflammatory genes, in addition to COX-2 and mPGES-1 [50]. The cellular localization of mPGES-1 in the present study, suggested a close connection to the cells expressing EP_1–2 _receptors, which also support an autocrine/paracrine regulatory pathway. An earlier study [35] showed the content of mPGES-1 in ovarian cancer cell lines, but the present study is the first, to our knowledge, to demonstrate the expression of mPGES-1 in EOC.

Previous studies have demonstrated that PGE_2 _acted mainly via the EP_2 _receptor in female reproduction and in tumourigenesis, while EP_1 _was more involved in neuronal functions [29]. Significant increases were found for the expressions of both EP_1 _and EP_2 _in AC compared to benign tumours in the present study. Interestingly, one of the findings in the present study was a difference in the distribution of EP-receptors between stroma and tumour tissue. The majority of the EP_1–2 _immunoreativity was found in OSE while only EP_2 _could be demonstrated in the stroma. First, this suggests target cells for PGE_2 _in both these cell compartments and secondly a separate function, mediated by the EP_2 _receptor in the stroma. In addition, the immunblotting experiment demonstrated multiple bands for the two receptors. These bands could represent phosphorylated forms of the receptors as well as splice variants [29], and therefore have important implications for cell signalling. The two receptors regulates different intracellular signalling pathways, i.e. IP_3_/Ca^2= ^(EP_1_) and cAMP (EP_2_). Phosphorylated residues and splice variants can potentially alter sensitivity, responsiveness and preferred signalling system within the cells [29], all of which can contribute to enhanced tumour growth. However, additional experiments are needed to explore these possibilities.

Staining of cluster of cells in the AC, demonstrated for primarily for mPGES-1 and EP_1–2_, were earlier reported for both COX-1 and COX-2 [36]. Previous studies have actually localised COX enzymes to a large variety of cells, including both normal and tumour epithelial cells, normal and tumour vascular endothelium and smooth muscle cells, macrophages and fibroblast cells. The clearly defined staining of populations of malignant cells, found here and by others, may implicate specific functions, such as angiogenesis and invasion or a more active anti-tumour response for mPGES-1 and EP_1–2 _in different populations of the malignant cells. Cluster like expression of COX-2 was recently reported to correlate to angiogenesis in prostate cancer [54].

## Conclusion

In the present study, statistically significant increases, as well as cell-specific localizations were demonstrated for COX-1, COX-2 and mPGES-1 in adenocarcinomas compared to the normal ovary and benign tumour tissue. These elevations in malignant cells can be a response to an inflammatory reaction, both chronic and acute, caused by a growing and potentially invasive tumour, and the interactions between tumor cells, stromal cells and the immune system.

The increase of COXs, mPGES-1 and EP receptors in epithelial ovarian tumours supports the hypothesis that PGE_2 _is an important factor for progression (proliferation/angiogenesis) in ovarian tumours. The appearance of COX-2 in potential pre-malignant structures (inclusion cysts) may indicate a role in malignant transformation of epithelial cells, in addition to its earlier documented presence in malignant tumours. The results also suggest that the synthesis of PGE_2 _is regulated by autocrine and paracrine factors (i.e. cytokines) as a result of epithelial-stromal interactions. Furthermore, mPGES-1 and the EP receptors may represent important targets for development of novel anti-inflammatory and anti-tumour therapies.
